# Utilization of ultrasonography to detect developmental dysplasia of the hip: when reality turns selective screening into universal use

**DOI:** 10.1186/s12887-017-0882-0

**Published:** 2017-06-05

**Authors:** Rachel Wilf–Miron, Jacob Kuint, Ronit Peled, Asaf Cohen, Avi Porath

**Affiliations:** 1 The Gertner Institute for Epidemiology and Health Policy Research, Sheba Medical Center, Ramat Gan, Israel; 2 The School of Public Health, Sackler Faculty of Medicine, Tel Aviv University, Tel Aviv, Israel; 3 Department of Neonatology, Edmond and Lily Safra Children’s Hospital, Tel Aviv, Israel; 4 Sackler Faculty of Medicine, Tel Aviv University, Tel Aviv, Israel; 5 Maccabi Healthcare Services, Tel Aviv, Israel; 6 Department of Health Systems Management, Faculty of Health Sciences, Ben-Gurion University of the Negev, Be’er Sheva, Israel; 7 Maccabi institute for Health Services Research, Maccabi Healthcare Services, Tel Aviv, Israel

**Keywords:** Quality of care, Cost, Hip developmental dysplasia, Ultrasonography

## Abstract

**Background:**

Developmental dysplasia of the hip (DDH) occurs in 3–5 of 1000 live births and is associated with known risk factors. In most countries, formal practice for early detection of DDH entails the combination of risk factor identification and physical examination of the hip, while the golden standard diagnostic instrument is hip ultrasonography (US). This practice is commonly referred to as selective screening. Infants with positive US findings are treated with a Pavlik harness, a dynamic abduction splint.

The objective of our study was to evaluate hip US utilization patterns in Maccabi Healthcare Services (MHS), a large health plan.

**Methods:**

Study population: All MHS members, born between June 2011 and October 2014, who underwent at least one US before the age of 15 months. Study variables: Practice specialty and number of enrolled infants. Positive US result was defined as referral to an abduction splint. Cost was based on Ministry of Health price list. Chi square and correlation coefficients were employed in the statistical analysis.

**Results:**

Of the 115,918 infants born during the study period, 67,491 underwent at least one hip US. Of these, 60.6% were female, mean age at performance: 2.2 months. Of those who underwent US, 625 (0.93%) were treated with a Pavlik harness: 0.24% of the male infants and 1.60% of the female infants (*p* < 0.001). Analysis of physician practice characteristics revealed that referral to US was significantly higher among pediatricians as compared with general practitioners (60% and 35%, respectively). Practice volume had no influence on referral rate. Direct medical costs of the 107 hip US examinations performed that led to detection of one positive case (treated by Pavlik): US$10,000.

**Conclusions:**

Current pattern of hip US utilization for early detection of DDH resembles universal screening more closely than selective screening. This can inform policy decisions as to whether a stricter selective screening or a formal move to universal screening is appropriate in Israel.

## Background

The term *developmental dysplasia of the hip* (DDH) describes a spectrum of conditions related to the abnormal development of the acetabulum and proximal femur leading to mechanical instability of the hip joint in infants and young children [1]. The prevalence of DDH varies from 1.6 to 28.5 cases per 1000 live births, depending on the definition and the population being studied. Most cases of DDH resolve without treatment in the first few months of life [2]. Bialik et al. suggested that “true DDH” incidence of hips with sonographic DDH that did not progress to normal and needed treatment throughout the 12 months of follow-up, is 5 cases per 1000 children [3]. DDH is more common among females compared with male infants, with a relative risk ratio of 2.54 [4]. The condition is also more common among infants with a positive family history or those experiencing abnormal positioning and/or limited fetal mobility, such as breech position [4, 5]. However, the majority of infants with symptomatic DDH evidence no risk factors: a systematic literature review reveals that, only 10–27% of all infants diagnosed with DDH in a population- based studies have identified risk factors (with the exception of female gender) [6–8].

The American Academy of Pediatrics recommends that all newborns be clinically examined for DDH in the first few days of life and at every health supervision visit until the child walks normally [9]. It should be noted that, neonatologists failed to detect about 50% of unstable hips in the initial examination [10]. In infants older than 3 months, unilateral limited hip abduction had a sensitivity of 69% and a specificity of 54% in the detection of ultrasonographically confirmed DDH [11]. Ultrasonography (US) is the diagnostic tool in infants with abnormal physical examination and in infants with risk factors. Until 4–6 months of age, US is the primary imaging technique used to assess the morphology and stability of the infant hip [12, 13]. At age 2 weeks to 6 months, dislocation or persistent instability are treated in Israel as elsewhere, with abduction devices, the Pavlik harness being most commonly used [14, 15]. Two types of screening can be performed: universal screening, in which all neonates are evaluated, and selective screening, in which only those at high risk are evaluated [16, 17]. Universal screening increases DDH detection, which leads to higher rates of treatment with abduction splinting; however, the universal screening approach may lead to high costs, unnecessary treatment, and increased post-treatment complications of avascular necrosis [18, 19] without, however, reducing the time required to accurately diagnose DDH. One should always bear in mind that late diagnosis increases treatment complexity and risks: In the short term - the need for prolonged hospitalization (accompanied by pain, inconvenience and the interruption of the child’s daily activities) and the risks of general anesthesia for both closed reduction or open reduction; recurrent dislocation and subluxation and osteochondritis. In the short-term, late diagnosis results in a sevenfold increase in the costs of treatment, compared to early detection and successful management in a Pavlik harness [20]. In the long term – increased risk of osteoarthritis and total hip replacement [21]. When the quality of the clinical examination is high, universal US screening has been found to be unnecessary [22]. The American Academy of Pediatrics thus recommends selective US screening for infants with risk factors (female infants born in the breech position, or those with a positive family history of DDH) or abnormal clinical examination findings [9]. US examinations in infants with clinically detected hip instability have been proven to reduce abduction splinting without increasing the rates of abnormal hip development or surgical treatment [12]. This policy was also found to reduce costs [23]. Yet, despite insufficient clinical evidence regarding US strategies, researchers believe that the optimum strategy is to use physical examinations to screen all neonates for hip dysplasia and use hip US selectively, for infants at high risk for DDH and infants with abnormal physical examination [17, 24]. In this scheme, commonly termed “selective screening”, US serves as a screening tool and a golden standard diagnostic instrument at the same time.

The Israeli Task Force on Health Promotion (last update on 2013) advocates US screening among infants with risk factors and infants with abnormal physical examination [25]. Ministry of Health instructions in Israel clearly state that US should be performed according to clinical indications and not as a universal modality [26]. The Ministry’s list of indications include: Clinical signs of hip joint instability, family history of DDH, breech delivery, oligohydramnios and musculoskeletal abnormalities related to tight intrauterine packing (foot or knee deformities, torticollis). The Israeli Task Force adds twin pregnancy and birth weight smaller than 2.5 kg or larger than 4.0 Kg.

Maccabi Healthcare Services (MHS), the second-largest health plan in Israel, provides primary and secondary community-based services to two million beneficiaries. This takes place under universal health insurance coverage that guarantees a universal “basket of services”, including US for the screening of DDH. Services are provided by MHS throughout the country, with a core staff of 8000 physicians, including 2000 primary-care physicians, 1000 nurses and other health professionals. Physicians are usually self-employed; they engage in 17 million physician–patient encounters annually. Every MHS member is allocated to a primary care physician who acts as his/her case manager. Primary care for infants and children is provided by pediatricians or general practitioners. In-patient care is purchased by MHS from local medical centers.

Recently, researchers found that 14% [27] and 19% [28] of the newborns were referred to hip US assessment due to clinical signs or risk factors. In the absence of data-based evidence, we hypothesized that screening US in Israel is performed at a higher rate than in other countries performing selective screening. The objectives of our study were: 1) to explore US referral patterns for DDH screening; 2) to study the variation between referral pattern and practice characteristics; and 3) to estimate the economic implications of these patterns.

## Methods

### Setting and study period

The study was conducted by MHS for the period between June 2011 and October 2014.

### Study population

All MHS members born between June 2011 and October 2014 and who had undergone at least one hip US before the age of 15 months.

### Data source

MHS is a fully computerized organization. Data on US examinations and Pavlik harness treatments was retrieved from MHS’s computerized billing systems. Our data did not include documentation of the reason for referral (i.e. signs of hip instability or mentioning of risk factors).

### Variables in the analysis

1) Volume of primary care practice from which the infant was referred, i.e., number of enrolled infants, aged 0–15 months, during the study period. Practices with less than 50 enrolled children were excluded from the analysis because small volume does not reflect referral patterns: The respective physicians might be new to MHS or in practice for very few hours weekly. 2) Physician’s specialty: general practice or pediatrics; 3) Infant’s gender and age at first US examination; 4) Positive US result, defined as referral for an abduction splint; 5) Cost of hip US, as indicated at the Ministry of Health price list, adjusted to January 2015. Data on indirect costs of hip US, such as cost of transportation to the medical facility or loss of parent’s work days when accompanying the infant to the examination could not be obtained and so were ignored.

### Statistical methods

Chi square tests were performed to evaluate differences in infant hip US referrals and practice characteristics. Correlation coefficients were calculated for practice volume and first referrals.

## Results

During the study period, 115,918 infants, members of MHS, were born, of which 51.6% were male and 48.4% female. Out of the study population, 67,491 (58.2%) underwent at least one US to detect DDH. Rates of hip US were higher among females than among males (60.6% and 56.0%, respectively; *p* < 0.001). The infants’ mean age at performance of the first hip US was 2.2 months (±1.28), being 2.21 (±1.24) for males and 2.23 months (±1.33) for females (*p* < 0.001). Of those who underwent hip US, 675 infants (0.93%) were diagnosed as positive for DDH and thereafter treated with the Pavlik harness. The proportion of positive DDH infants requiring a harness was higher among females than among males: 1.60% and 0.24% respectively (*p* < 0.001) (Table 1). The 625 infants requiring a harness represent a crude overall treatment rate of 5.39 per 1000 live births.

**Table 1:** Study population characteristics

Variable	Total	Male	Female	*P* Value
Infants (*N* = 115,918)				
Birth cohort	115,918	59,779 (51.6%)	56,139 (48.4%)	--
Mean age (months)	2.2 (±1.28)	2.21 (±1.24)	2.23 (±1.33)	<0.001
Initial US^a^	67,491 (58.2%)	33,472 (56.0%)	34,019 (60.6%)	<0.001
Positive US	625 (0.93%)	81 (0.24%)	544 (1.60%)	<0.001
Physicians (*N* = 487)				
	General Practitioners (*N* = 50)	Pediatricians (*N* = 437)		
Practice volume	5110 (4.4%)	110,289 (95.6%)		--
Mean practice volume	100 (±58.5)	252 (±185.5)		--
Initial referral	1790	65,701		--
Referrals as a proportion of practice volume	35.0%	59.6%		<0.001
Positive US	19 (1.06%)	606 (0.92%)		0.631

^a^ *US* = ultrasonography

Among the 487 physicians who referred newborns for hip US and thus included in the analysis, 437 were pediatricians with 110,289 registered infants during the study period; the remaining 50 physicians were general practitioners (GPs) with 5110 registered infants during the same period. The mean practice volume of infants in pediatric and GP clinics was 252 (±185.5) and 100 (±58.5), respectively. The number of infants referred by pediatricians and GPs for hip US was 65,701 and 1790, respectively. Those referrals constituted 59.6% and 35.0% of registered infants in the pediatric and general practices, respectively. US proved positive in 0.92% and 1.06% of referrals in pediatric and general practices, respectively (*p* = 0.631) (Table 1).

**Fig. 1:**
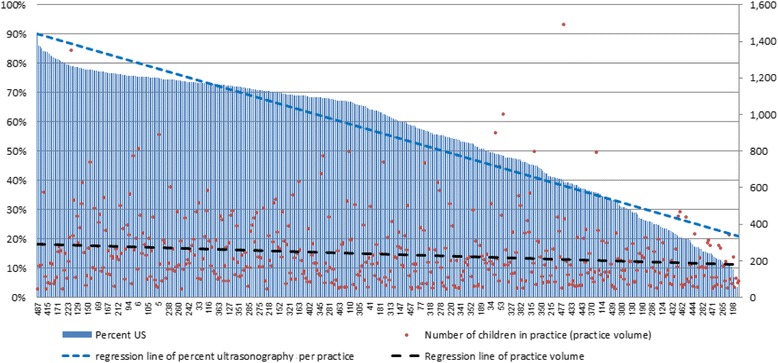
Practice volume and first US referrals

Figure 1 demonstrates a positive but weak correlation between volume of practice and referral rate to first hip US (*r* = 0.182; *p* < 0.001).

The cost of each hip US for early detection of DDH, in Israeli prices (NIS), based on the official Ministry of Health price list is NIS 361 (US$ 94). In terms of the health plan, 107 examinations were performed with only one case diagnosed as requiring a Pavlik harness (positive case). Hence, the total cost of detecting one case of DDH during the study period reached NIS 38,627 or US$ 10,016.

## Discussion

This study demonstrates high utilization of hip US to detect DDH among Israeli physicians. Our Ministry of Health and local professional associations have clearly recommended the selective screening approach, meaning a referral to US following a positive physical examination or high risk indication. Despite this recommendation, a detailed “gold standard” indicating “appropriate” utilization of sonography in selective screening of infant hip DDH has yet to be clearly defined.

Laborie reported the results of 16 years of implementing the selective US strategy, with findings suggesting that although 14% of all newborns were defined “at risk” and referred to hip US, only 3% of these infants received early treatment [27]. Clarke et al. [28] analyzed a prospective cohort of 107,000 live births and found that whereas 19% were referred to hip US assessment due to clinical signs or risk factors, only 3.8% were diagnosed with dysplasia, a crude overall rate of 7.2 cases per 1000 live births. Over the 20-year-study period, the rate of referrals to hip US increased by 5% annually, although the rate of Pavlik harness treatment remained stable [28].

With respect to practice characterization, our study found that the volume of infants registered in the physician’s practice had little influence on referral patterns. However, pediatricians demonstrated significantly higher referral rates when compared with GPs. We do not have data-based explanation for this finding. In the absence of specific data, we may suggest that practices concerning US utilization may also differ by specialty in other countries.

Our data indicates nearly 60% of the infants born during the study period underwent hip US examination during the first 15 months of life. This rate is three times higher than the cited UK and Norwegian data [27, 28]. The rate of treatment in our study was nonetheless similar to those found in the literature, which may be explained by the low rate (0.93%) of positive findings in hip US, a fact that “corrects” for the high rate of hip US examinations. The high rate of first referrals and very low positive diagnosis rate thus demonstrate non-adherence to national guidelines, what might contribute to this significantly high level of imaging.

The literature from the last decade has been conflicting: For example, a recent Cochrane analysis has indicated that "there is insufficient evidence to give clear recommendations for practice.... Neither of the ultrasound strategies has been demonstrated to improve clinical outcomes including late diagnosed DDH and surgery" [24]. The conflicting evidence may contribute to confusion and non-adherence. Furthermore, the fact that hip US is included in Israel’s basic basket of services means that the examination is provided “gratis” to all citizens. Pricing issues therefore do not create barriers to US overuse. In addition, hip US is a non-invasive, safe technology that imposes little inconvenience upon infants or parents. Since health plan members are increasingly knowledgeable and active consumers, parents may be applying pressure on physicians to refer newborns to the examination in order to rule out any possibility – however remote – of DDH. As pre-authorization is not required for the hip US, there is no counter-pressure to limit referrals.

Therefore, the decision to refer an infant for screening rests on the subjective judgment of the primary care physician. Primary care physicians may also be aware of the limitation of the physical examination for hip instability and the far-reaching consequences of late-detection for patients. For that reason they might prefer a more valid screening method like US.

The frequency of claims regarding misdiagnosis of DDH in childhood have greatly declined in recent years, probably due to advances in US technology [29]. In Israel, very few claims have been filed during the last 20 years (based on unpublished data of Israel’s leading professional liability insurance provider). In the absence of data on the incidence of late-detected DDH cases in Israel, the claim filing data might suggest that this phenomenon is relatively rare.

Measurement is an essential first step toward encouraging more appropriate use of imaging. US screening for DDH at a rate close to 60% imposes a considerable burden in terms of unnecessary direct costs, with two-thirds of the imaging probably unwarranted. Also to be considered are the reduced national productivity levels caused by parents absenting themselves from work in order to accompany the infant; exaggerated anxiety regarding a possible diagnosis of DDH; together with the potential over-treatment and complications due to false positive results of the hip US.

This study, the first conducted by MHS to evaluate patterns of hip US utilization, demonstrates a pattern which resembles universal screening more closely than selective screening. This gap between national recommendation and the actual practice invites policy makers to re-evaluate the current situation and decide whether a stricter selective screening or formal move to universal screening is appropriate in Israel. Until a formal change in the national policy (which might take quite a long time), we suggest a number of steps that might be taken: refreshment of guidelines in tandem with discussions of uncertainty and other clinical and organizational issues; distribution of personal referral patterns among practicing physicians; and redefinition of referral patterns in the form of organizational quality measures while setting annual targets.

Our analysis nevertheless exhibits some limitations: 1) The referral data was retrieved from the MHS billing system, which allows calculation of rates of performance but not analysis of the reasons for the referral (e.g., abnormal clinical findings or the presence of risk factors); 2) The cost data reflects only the known cost of performing a hip US; other direct costs, such as additional physician encounters, or indirect costs, such as loss of productivity, transportation expenses or the long-term consequences of overuse, are not captured by this variable.

## Conclusions

Current pattern of hip US utilization for early detection of DDH resembles universal screening more closely than selective screening. This can inform policy decisions as to whether a stricter selective screening or a formal move to universal screening is appropriate in Israel.

## Abbreviations

DDH: Developmental dysplasia of the hip
GP: General practictitioner
MHS: Maccabi Healthcare Services
NIS: New Israeli shekel
US: Ultrasonography
